# 

**Published:** 1908-02-29

**Authors:** 


### THE GRADUATES' MEDICAL SCHOOLS.

### Diary for the week March 2 to 7.

### THE ROYAL COLLEGE OF SURGEONS.

**At 5 p.m.**

**March 2, 4, and 6,** Prof. F. C. Shrubsall, **The Physical Anthropology of the Pigmy and Negro Races of Africa.**

### THE HOSPITAL FOR SICK CHILDREN, Great Ormond Street, W.C.

**At 4 p.m.**

**March 5,** Dr. Hutchison, **Intestinal Worms and their Treatment.**

### NATIONAL HOSPITAL FOR THE PARALYSED AND EPILEPTIC, Queen Square, Bloomsbury, W.C.

**At 3.30 p.m.**

**March 3,** Mr. Ballance, **Surgery of the Nervous System.**
**March 6,** Mr. Armour, **Surgery of the Nervous System.**

### MEDICAL GRADUATES' COLLEGE AND POLYCLINIC, Chenies Street, W.C.

**Lectures at 5.15 p.m.**

**March 2,** Dr. G. H. Graham, **Photo-Therapy.**
**March 3,** Dr. G. E. Herman, **The Use of the Curette.**
**March 4,** Prof. W. E. Dixon, **The Action of Tobacco Smoke.**
**March 5,** Dr. Dundas Grant, **Acute Inflammation of the Middle Ear.**

### THE POST-GRADUATE COLLEGE, West London Hospital, Hammersmith, W.

**At noon.**

**March 2,** Dr. Low, **Pathological Demonstration.**
**March 3 and 5,** Dr. Pritchard, **Practical Medicine.**

**At 5 p.m.**

**March 2,** Mr. Dunn, **Cases of Eye Disease.**
**March 3,** Dr. Seymour Taylor, **Chlorosis—Gastric Ulcer.**
**March 4,** Mr. Paget, **Clinical Lecture—I.**
**March 5,** Mr. Baldwin, **Practical Surgery—IV.**
**March 6,** Mr. Paget, **Clinical Lecture—II.**

### LONDON SCHOOL OF CLINICAL MEDICINE, Seamen's Hospital, Greenwich.

**At 4 p.m.**

**March 2,** Dr. St. Clair Thomson.

**At 2.15 p.m.**

**March 4,** Dr. F. Taylor, **Congenital Heart Disease.**

### THE THROAT HOSPITAL, Golden Square, W.

**At 5.30 p.m.**

**March 2.** Mr. T. J. Faulder, **Diseases of the Perceptive Mechanism—Acute.**
**March 5,** Mr. T. J. Faulder, **Diseases of the Perceptive Mechanism—Chronic.**

### CHARING CROSS HOSPITAL, W.C.

Post-Graduate Course.

**At 4 p.m.**

**March 5,** Mr. Fairbank, **Othopædic.**

### THE HOSPITAL FOR NERVOUS DISEASES, Welbeck Street, W.

**At 5 p.m.**

**March 5,** Dr. Harry Campbell. **Selected Cases.**

### CENTRAL LONDON THROAT AND EAR HOSPITAL, Gray's Inn Road, W.C.

**At 4 p.m.**

**March 5,** Mr Chichele Nourse, **Antral Suppuration.**

### HOSPITAL FOR CONSUMPTION AND DISEASES OF THE CHEST, Brompton, S.W.

**At 4 p.m.**

**March 4,** Dr. Wethered, **Pulmonary Œdema.**

### MOUNT VERNON HOSPITAL, Fitzroy Square.

Post-Graduate Course.

**At 5 p.m.**

**March 5,** Mr. James Berry, **Recent Advances in the Surgery of the Chest.**

### BOOKS RECEIVED.

H. K. Lewis.

"An Essay upon Disease; Its Cause and Prevention." By G. E. Richmond, M.D.

Edward Arnold.

"The Chemical Basis of Pharmacology." By Francis Francis, D.Sc., and J. M. Fortescue-Brickdale, M.D.

J. Willing, Jun., Ltd.

"Willing's Press Guide, 1908."

We have received from Bovril, Ltd., a large handsomely framed artist proof of the picture by Arthur Elsley, entitled "Well Done." The shock-headed pony, which forms the most striking figure in the group, is particularly good, and the gravure reproduction by Faulkner and Co. is soft and pleasing. Purchasers of Bovril can obtain prints of this charming picture in accordance with the published conditions of the "Bovril Picture Scheme."

JUST READY. 5th Edition, 30th Thousand. Revised and Enlarged. 234 Illustrations. Limp cloth, with flap, 2/6 net; or stiff paper, 1/- net.

**"FIRST-AID" TO THE INJURED AND SICK:**

**AN ADVANCED AMBULANCE HANDBOOK.**

By F. J. WARWICK, B.A., M.B.(Cantab.), M.R.C.S., L.S.A., and A. C. TUNSTALL, M.D., F.R.C.S.(Edin.).

"Has already taken its place as a standard work."—*Brit. Med. Jour.* "Now one of the most satisfactory of all text-books on 'First Aid.'"—*Glasgow Med. Jour.* "Undoubtedly one of the most comprehensive text-books yet issued."—*First Aid.*

4th Edition. On Sheets 3 ft. 4 in. by 2 ft 2 in., 2/- each; or 27/6 net the set of 18, with nickel heads for suspension; or mounted on linen, 45/- net.

**FIRST AID WALL DIAGRAMS:**

**BEING ENLARGEMENTS OF THE ABOVE ILLUSTRATIONS.**

ADOPTED BY THE WAR OFFICE, THE ADMIRALTY, LONDON SCHOOL BOARD, &c.

*Full Illustrated Prospectus on application.*

6th Edition. Revised and Enlarged. Many Illustrations. 7/6 net.
**LECTURES ON MASSAGE & ELECTRICITY**
**IN THE TREATMENT OF DISEASE.**
By THOS. STRETCH DOWSE, M.D.
"A most interesting and practical book."—*Scott. Med. and Surg. Jour.*
**Bristol: JOHN WRIGHT & CO.**

On Sheets 2 ft. 2 in. by 3 ft. 4 in., 2/- each, or 42/- the set of 26 sheets, with Nickeled Head for suspension; or mounted on linen, 68/-
A SERIES OF
**MIDWIFERY DIAGRAMS:**
**FOR THE INSTRUCTION OF MIDWIVES AND STUDENTS OF MIDWIFERY.**
By VICTOR BONNEY, M.S., M.D., B.Sc. (Lond.), F.R.C.S., M.R.C.P., *Lecturer on Practical Midwifery, Middlesex Hospital.*
The figures (160 in number) constitute a complete pictorial course in the subject as far as applicable to those for whom they are designed.
*A full Illustrated Prospectus on application.*

3rd Edition Revised. Pocket Size. 1/- net.
**A GUIDE TO URINE TESTING: FOR NURSES.**
By MARK ROBINSON, L.R.C.P. & S. Edin.
"An excellent little guide—short, concise, and accurate."—*Dub. Med. Jour.*

2nd Edition. 1/-. Pocket Size.
**GOLDEN RULES OF SICK NURSING.**
By W. B. DRUMMOND, M.B., C.M., F.R.C.P. Edin., *Assist. Phys. at Roy. Hosp. for Sick Children, Edinburgh.*
"From the first page to the last it is full of sound teaching."—*Hospital.*
**London: SIMPKIN & CO., Ltd.**

